# Comparison of neonatal outcomes between category-1 and non-category-1 Primary Emergency Cesarean Section: A retrospective record review in a tertiary care hospital

**DOI:** 10.12669/pjms.344.14496

**Published:** 2018

**Authors:** Dur-e-shahwar, Iffat Ahmed, Azra Amerjee, Zahra Hoodbhoy

**Affiliations:** 1 Dr. Dur-e-Shahwar, FCPS. Department of Obstetrics and Gynaecology, The Aga Khan University Hospital, Karachi, Pakistan; 2 Dr. Iffat Ahmed, FCPS. Department of Obstetrics and Gynaecology, The Aga Khan University Hospital, Karachi, Pakistan; 3 Dr. Azra Amerjee, FCPS; MCPS HPE; PGD Bioethics. Department of Obstetrics and Gynaecology, The Aga Khan University Hospital, Karachi, Pakistan; 4 Zahra Hoodbhoy, Department of Obstetrics and Gynaecology, The Aga Khan University Hospital, Karachi, Pakistan

**Keywords:** Primary Emergency Caesarean section, Category-1 caesarean section, Fetal distress, Non-progress of labour, Ante-partum hemorrhage and malpresentation

## Abstract

**Objective:**

To compare neonatal outcomes between Category-1 and Non-Category-1 Primary Emergency Cesarean Section.

**Methods:**

This was a retrospective analysis, conducted at Aga Khan University Hospital Karachi from January 1^st^ 2016 till December 31^st^ 2016. Non-probability purposive sampling technique was used. A sample size of 375 patients who had primary Emergency Caesarean Section (Em-CS) was identified by keeping CS rate of 41.5% and 5% bond on error. Data was collected from labor ward, operating theatre and neonatal ward records by using structured questionnaire.

**Results:**

In the current study, out of 375 participants who underwent primary Em-CS; majority (89.3%) were booked cases. Two-hundred-eighty-two (75.2%) were primiparous women. Two hundred and thirty (61.3%) were at term and 145(38.7%) were preterm. The main indication among Category-1 CS was fetal distress (15.7%). For Non-Category-1 CS, non-progress of labour (45.1%) was the leading cause of abdominal delivery. Except for APGAR score at one minute (p value = 0.048), no other variables were statistically significant when neonatal outcomes were compared among Category-1 and Non-Category-1 CS.

**Conclusion:**

In this study, fetal distress and non-progress of labor were the main indications for Category-1 and Non-Category-1 CS respectively. We did not find statistically significant association between indications of Em CS and neonatal outcomes. However further prospective studies are required to confirm this association.

## INTRODUCTION

Overall cesarean section (CS) rate is rising worldwide over the past three decades. Wide variations remain at regional, national, and sub-national level.^1^ Rapid increase in Caesarean delivery rate has become a serious public health issue in both developed and developing countries. One of the important indicators of the availability of quality obstetric care of any obstetric unit is the proportion of CS to total births (WHO 2009). WHO suggests optimum CS rate of around 15%.^2^ Latest data (1990-2014) from 150 countries showed mean CS rates ranging from 3.5% in sub-Saharan Africa to 40.5% in Latin America and the Caribbean.^3^ In Pakistan, CS rate is reported significantly high among primigravid women (27.26%).^4^

Wide variation in CS rates is related to clinical and non- clinical factors.^5^,^6^ Clinical factors include low parity, extremes of reproductive age, height less than 150 cm, obesity, use of cardiotocography (CTG), fetal mal-presentation and low fetal birth weight.^7^,^8^ Private hospital status, organizational factors, woman’s choice regarding childbirth, and the obstetrician’s characteristics are few of the non-clinical factors.^9^

The Obstetrics and Gynecology unit in the Aga Khan University Hospital (AKUH) is a busy unit with 4500-5000 deliveries per year. Primary emergency caesarean section (Em-CS) is a performance indicator of our unit because this group not only determines the future obstetric course of a woman but has a major impact on institutional statistics of CS. Em-CS as against elective caesarean section (El-CS) is often related to maternal and neonatal complications. Amongst Em CS, Category-1 CS is more frequently associated with adverse neonatal outcomes than other indications of Em CS (Non-Category-1 CS).^10^,^11^

The rationale of this study was to compare neonatal outcomes between Category-1 and Non-Category-1 Primary Em-CS.

## METHODS

This was a retrospective study to compare neonatal outcomes between Category-1 and Non-Category-1 Primary Em-CS performed after 24 weeks of gestation in the Department of Obstetrics & Gynecology AKUH from January 2016 till December 2016.

Total of 375 patients (both term and preterm), who underwent primary Em-CS were included using non-probability purposive sampling technique. This sample size was calculated by keeping C-section rate of 41.5% and 5% bond on error.^12^ Discrimination between women in spontaneous labour and those being induced was not considered. Data was collected on a pre-tested structured questionnaire, after approval from hospital Ethical Review Committee (ERC).

Variables included maternal socio-demographic features, complications during pregnancy, and indications for CS. Neonatal outcomes included APGAR scores at one and five minutes, birth weight (kg), cord pH, admission of baby to NICU and early neonatal death.

Indications for Em-CS were categorized in Category-1 and Non-Category-1 CS groups. These indication included failure to progress in labor, fetal distress, pre-eclampsia, antepartum hemorrhage (APH), malpresentation, intrauterine growth restriction (IUGR) and twins with preterm labor.

Data was entered and analyzed using SPSS version 19. Categorical variables were reported as proportions and continuous variables as means and standard deviations. Univariate analysis was done using chi-square test for categorical variables and student t-test for continuous variables. P-value of < 0.05 was considered significant.

### Operational Definitions

- **Categories of Caesarean Section:**Indications of CS are classified into four categories depending on the degree of urgency of caesarean section related to the immediate risks to mother or fetus.^13^ We classified the indications into two broad categories (Category-1 and Non-Category-1). Category-1 included all indications that posed an immediate risk to the life of the mother or fetus (e.g pathological trace CTG, abruptio placentae) and required a decision-to-delivery interval of not more than 30 minutes. Non-Category-1 (Category 2 and 3) included cesarean sections performed for indications that posed no immediate threat to life of woman or fetus but required early delivery. We excluded Category-4 CS that includes CS performed at a time to suit the woman and maternity services (Elective Cesarean Section) and where there is no maternal or fetal compromise.
- **Fetal Distress:** included all cases where the CTG showed either a pathological pattern or a suspicious trace along with other risk factors IUGR, preeclampsia, placenta previa, placental abruption or preterm labour.^14^
- **Early Neonatal Deaths:**included deaths of neonates within seven days of birth.,
- **Primary Caesarean Section:**is the woman’s first caesarean delivery, even if she has given birth vaginally before.

## RESULTS

Out of a total of 375 participants who underwent primary Em-CS, majority (89.3%) were booked cases. Two-hundred-eighty-two (75.2%) were primiparous. (Table-I) In the current study, the commonest indications for primary emergency cesarean section were non-progress of labor, fetal distress, preeclampsia and placental abruption. (Table-II) Two hundred and thirty (61.3%) were at term and 145(38.7%) were preterm deliveries. Out of 145 preterm cases, 60 newborns were admitted in NICU of which 13 (3.5%) were neonatal deaths (NND). (Table-IV) Other NICU admissions included term deliveries with neonatal hyperbilirubinemia (n=6, 7.5%), hypoglycemia (n=6, 7.5%), sepsis (n=4, 5%) and respiratory distress syndrome (n=3, 3.7%).

**Table-I:** Maternal demographics of all participants.

Variables	Participants who underwent primary emergency cesarean section (N=375) Mean +/- SD
Age (years)	27.9 +/- 4.7
Weight (kg)	68.8 +/- 13.5
Height (cm)	157.1 +/- 9.1
BMI (kg/m^2^)	27.7 +/- 5.6
Booking status*	335 (89.3)
Parity*	
Primiparous	282 (75.2)
Multiparous	92 (24.6)
Grand-multiparous	1 (0.3)
Term pregnancies	230 (61.3)
Preterm pregnancies	145 (38.7)

* Reported as N (%); BMI: Body Mass Index

**Table-II:** Indications for Cesarean section*

Indications for Cesarean section	Participants who underwent primary emergency cesarean section n (%)
Category-1 Cesarean section	
Fetal distress	59(15.7)
Placental abruption	34(9.1)
Non-Category-1 Cesarean section	
Non progress of labor	169(45.1)
Preeclampsia	36(9.6)
Fetal malpresentation	33(8.8)
IUGR	28(7.5)
Preterm labor	16(4.3)

IUGR – Intrauterine Growth Restriction

**Table-III:** Comparison for complications during pregnancy and indications of C/S in all pregnancies.

Variables	Category 1 C/S (N=93)	Non-Categories 1 C/S (N=282)	p-value
Age (years)	28.8 +/- 5.1	27.7 +/- 4.5	0.05
Weight (kg)	67.8 +/- 14.1	69.1 +/- 13.3	0.42
Height (cm)	157.3 +/- 5.5	156.9 +/- 9.9	0.71
BMI (kg/m^2^)	27.4 +/- 5.6	27.8 +/- 5.6	0.57
Booking status*	80 (86)	255 (90)	0.23
Parity*			0.06
Primiparous	59 (63.4)	223 (79.1)	
Multiparous	34 (36.6)	59 (20.9)	
Term	50 (53.8)	180 (63.8)	0.06
Preterm	43 (46.2)	102 (36.2)	
Complications during pregnancy*			<0.001
Hypertension in pregnancy	6 (13.3)	47 (48)	
Gestational diabetes	2 (4.4)	10 (10.2)	
Placenta previa/abruption	30 (66.7)	0 (0)	
SGA/IUGR	4 (8.9)	20 (20.4)	
Others	3 (6.7)	21 (21.4)

**Table-IV:** Comparison of Perinatal outcomes in women with Category 1 CS (fetal distress and placental abruption) compared to other causes for CS (Non-Category 1 CS).

Variables	Indication Category 1 CS (N=93)	Indication Non-Categories 1 CS (N=282)	p-value
Gestational age at delivery (weeks)	36.4 +/- 3.1	36.7 +/- 3.2	0.39
APGAR score at 1 minute	7.6 +/- 1.1	7.8 +/- 0.8	0.048
APGAR score at 5 minutes	8.8 +/- 0.9	8.9 +/- 0.6	0.25
Birth weight (kg)	2.6 +/- 0.7	2.6 +/- 0.8	0.51
Cord pH	7.2 +/- 0.07	7.2 +/- 0.08	0.49
Perinatal outcomes*			
-Alive	90 (96.8)	272 (96.4)	0.88
- Neonatal death	3 (3.2)	10 (3.6)	
Admission of baby*			
-Well baby	70 (75.3)	226 (80.1)	0.32
-NICU	23 (24.7)	56 (19.9)	

* Reported as N (%)

Except for APGAR score at one minute (p value = 0.048), no other variables were statistically significant when perinatal outcomes was compared among Category-1 and Non-Category-1 CS.

## DISCUSSION

In this study, perinatal outcomes were not statistically significant among women who underwent Category-1 primary Em-CS compared to other causes (Non-Category-1 CS), except for APGAR score at one minute. Fetal distress and placental abruption were the indications for Category -1, while non-progress of labor and preeclampsia were the two major indications for Non-Category-1 CS.

In our study, the demographic characteristics including age, BMI, and booking status were comparable in both categories. Two hundred and eighty two (75.2%) primary Em-CS were performed in primigravid women, 92 (24.6%) in multigravidas, while 1(0.3%) in grand-multiparas (Table-I). These findings suggest that primary emergency caesarean section is commoner in primiparous compared to multiparous women. This may be because labor is usually faster and smoother in the latter and these results are similar to Jain M et al.^15^

When we compared the rate of antenatal complications between Category-1 and Non-Category-1 CS groups, the difference was statistically significant. In Category-1 CS group the commonest antenatal complications were placenta previa and placental abruption. Whereas hypertensive disorders (48%) and IUGR (20.4%) complicated pregnancies in women who underwent Non-Category-1 CS.^15^

The most common indications (Table-I) for Category-1 CS in our study were fetal distress (15.7%) and placental abruption (9.1%); while Non-Category-1 CS were performed mainly for non-progress of labor (45.1%). Similar result was found by Singh G et al.^16^

CS for fetal distress was 59 (15.7%) in our study which is almost comparable to Chu K et al.^17^ Interpretation of CTG finding is highly subjective and strongly influenced by obstetric practice. Continuous electronic monitoring of the fetal heart has not been seen to necessarily improve perinatal outcome but increases the risk of caesarean section and instrumental delivery.^18^ A meta-analysis showed that continuous CTG monitoring supplemented with ST analysis may significantly reduce the total operative delivery rate (0.93; 0.88-0.99).^19^

Preeclampsia is a multisystem disorder, affecting pregnancy after 20 weeks of gestation. It may complicate pregnancy with placental abruption and/or placental insufficiency leading to IUGR, with progressive deterioration in both maternal and fetal conditions. Delivering the fetus is the only way to arrest the disease.^20^ In our study 36(9.6%) pregnancies were terminated for severe preeclampsia while placental abruption 34(9.1%) and IUGR 28 (7.5%) were other indications for Em-CS.^21^,^22^

Fetal mal-presentation 32 (8.5%) accounted for a significant number of Caesarean sections in our study. External Cephalic Version (ECV) at 36 weeks of gestation may be used as an intervention to reduce high caesarean section rate for breech presentation. However, ECV requires skills and might not always be successful.^23^

Out of 16 patients admitted with preterm labor majority (n=12) were twins. Data regarding the indications for CS in the remaining four patients admitted with preterm labour, was missing.

Perinatal outcomes among women who underwent Category-1 primary Em-CS compared to other causes (Non-Category-1 CS), showed no statistically significant difference except for APGAR score at one minute (p value = 0.048) (Table-II). Grace L et al have found poor perinatal outcome in Category-1 CS compare to Non-Category-1 CS, with longer duration of study (seven years) and larger sample size.^23^

Cause for NICU admission was prematurity in 75% of the cases. This does not directly correlate with the indication for Em-CS; rather it’s a confounding effect of gestation. Neonatal deaths were reported in 13 (3.5%) cases. The cause of poor perinatal outcome in these babies was extreme prematurity.

### Limitations of study

It was a retrospective study, conducted in single tertiary center on a relatively small sample size.

## CONCLUSION

Fetal distress and placental abruption were the indications for Category-1, while non-progress of labor and preeclampsia were the two major indications for Non-Category-1 CS. There was no statistically significant association between different indications of Em CS and neonatal outcome. Larger prospective studies are required to confirm/refute this association.

### Authors’ Contribution

***Dur-e-Shahwar:*** Conceptualization of topic, literature search, study design, data collection, analysis, results, write up and proof reading.

***Iffat Ahmed and Azra Amerjee:*** Conceptualization of topic, study design, data analysis, results, write up and proof reading.

***Zahra Hoodbhoy:*** Data analysis, interpretation and results.
